# Imidazole Compounds for Protecting Choroidal Endothelial Cells from Complement Injury

**DOI:** 10.1038/s41598-018-31846-z

**Published:** 2018-09-06

**Authors:** Shemin Zeng, Kuo-Kuang Wen, Grefachew Workalemahu, Elliott H. Sohn, Meng Wu, Kathleen R. Chirco, Miles J. Flamme-Wiese, Xiuying Liu, Edwin M. Stone, Budd A. Tucker, Robert F. Mullins

**Affiliations:** 1 The University of Iowa Institute for Vision Research, Iowa City, USA; 2 University of Iowa Department of Ophthalmology and Visual Sciences, Iowa City, USA; 3 University of Iowa Department of Biochemistry, Iowa City, USA

## Abstract

Age-related macular degeneration (AMD) is a common, blinding disease associated with increased complement system activity. Eyes with AMD show elevated accumulation of the membrane attack complex (MAC) in the choriocapillaris and degeneration of macular choriocapillaris endothelial cells (ECs). Thus, one could reasonably conclude that the endothelial cell death that occurs in AMD is due to injury by the MAC. We therefore sought to identify strategies for protecting ECs against MAC lysis. RF/6A endothelial cells were pre-incubated with a library of FDA-approved small molecules, followed by incubation with complement intact human serum quantification of cell death. Two closely related molecules identified in the screen, econazole nitrate and miconazole nitrate, were followed in validation and mechanistic studies. Both compounds reduced lysis of choroidal ECs treated with complement-intact serum, across a range of doses from 1 to 100 µM. Cell rescue was confirmed in mouse primary choroidal ECs. Both exosome release and cell surface roughness (assessed using a Holomonitor system) were reduced by drug pretreatment in RF/6A cells, whereas endosome formation increased with both drugs, consistent with imidazole-mediated alterations of cell surface dynamics. The results in the current study provide further proof of principle that small molecules can protect choroidal ECs from MAC-induced cell death and suggest that FDA approved compounds may be beneficial in reducing vascular loss and progression of AMD.

## Introduction

Age-related macular degeneration (AMD) is a common disease of the elderly in which the central visual field—responsible for daily tasks requiring high visual acuity—becomes compromised due to damage in the central, macular region of the retina. The impact of AMD on the lives of affected patients includes impairment in ability to read and recognize faces^1,2^ and is associated with depression and decreased quality of life^3,4^. The prevalence of AMD in Caucasians ≥85 years of age is as high as 17–27% (9.8–13.4% for advanced AMD), with increasing prevalence and severity with increasing age^5,6^.

Current therapies for AMD include over the counter vitamin supplements (Age Related Eye Disease Study, or “AREDS2”, vitamins) which have shown a 25% risk reduction in progression to the neovascular form of AMD patients over 5 years^7,8^. While their physiological function is not fully explored, this supplement, which includes antioxidants and zinc oxide in the current formulation, are effective in human organ culture at attenuating endothelial cell activation^9^. For patients with advanced neovascular AMD, in which choroidal and/or retinal blood vessels invade the normally avascular spaces beneath the neural retina or retinal pigment epithelium, agents that interfere with vascular endothelial growth factor (VEGF) are very effective at resolving fluid and, at least temporarily, inducing regression of the abnormal blood vessels in a majority of affected patients^10–12^. The therapeutic management of atrophic AMD, however, remains highly limited.

**Figure 1:**
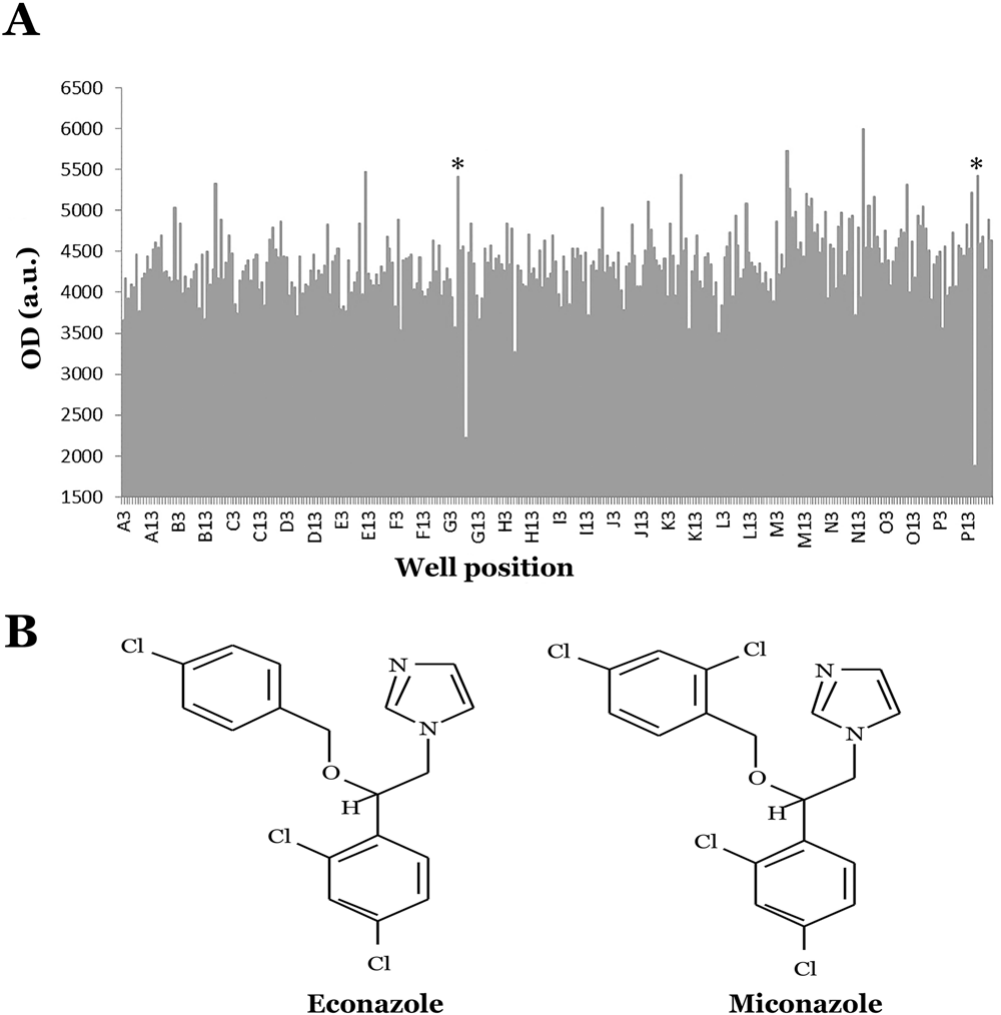
Drug screen results. (**A**) Example of positive hits (asterisks y-axis indicates cell survival using PrestoBlue). While some peaks appear higher in this experiment, two peaks that replicated across plates were selected for further study. (**B**) Structure of econazole and miconazole, two compounds that showed rescue in replicate plates and were chosen for further analysis.

One major insight into the pathogenesis of AMD, which may provide avenues for therapy, is that the terminal complement cascade becomes activated in domains surrounding the choriocapillaris, the blood supply for the retinal pigment epithelium and photoreceptor cells. Polymorphisms in genes involved in complement regulation are strongly associated with AMD and the membrane attack complex (MAC) of complement accumulates in the extracellular matrix surrounding choriocapillaris endothelial cells and, in some cases, on the surfaces of the endothelial cells themselves (Fig. 1) (recently reviewed by Whitmore *et al*.^13^).

The finding that MAC accumulates in aging choriocapillaris, and especially in AMD, in addition to morphometric studies showing loss of choriocapillaris endothelial cells in AMD prior to the loss of RPE or photoreceptor cells^14–16^ led us to develop a model of EC injury *in vitro*. In this model, the nonhuman primate endothelial cell line RF/6A, incubated with human complement-deficient (i.e., heat inactivated, C5-depleted or cobra venom factor-depleted) or complement-intact serum, was evaluated in terms of global gene expression, MMP9 synthesis, monolayer permeability, and cell death. Notably, increasing concentrations of complement-intact serum lead to endothelial cell lysis^17^.

A major advantage of modeling and quantifying complement-induced choroidal endothelial cell death in an immortal cell line *in vitro* is that it allows upscaling and testing of compound libraries. In the current study we describe a drug screen in which a library of FDA approved compounds was tested for its ability to protect RF/6A cells from complement-mediated lysis, and we describe two closely related compounds that show efficacy in rescuing ECs from cell death.

## Materials and Methods

### Cell culture

RF/6A cells (American Type Culture Collection, Manassas Virginia) were used for most experiments. We previously validated these cells in our collection as having the expected primate derivation by analysis of the *ICAM1* gene sequence^17^. Cells were maintained on T75 tissue culture flasks in DMEM with 10% fetal calf serum (Gibco, Gaithersburg, MD) with 1% penicillin-streptomycin (Gibco, Gaithersburg, MD) and passaged when ~90% confluent.

In addition, primary cultures of mouse choroidal endothelial cells were generated from F1 offspring of TIE2-GFP mice (Tg(TIE2GFP)287Sato/J) and albino C57Bl/6J mice (B6(Cg)-Tyrc-2J/J), both from the Jackson Laboratory (Bar Harbor, ME). All animal procedures were performed with approval by the University of Iowa’s Institutional Animal Care and Use Committee (IACUC) and in accordance with the ARVO statement on the use of animals. Briefly, 3 weeks old mouse were euthanized and the cornea, lens, iris and retina were separated from posterior eyecups. Eyecups containing choroid and sclera from 10 eyes (5 mice) were chopped into ~2 mm squares and digested with 4x digestion buffer: 25 mg/mL collagenase A (Sigma-Aldrich), 25 mg/mL dispase II (Sigma-Aldrich), 250 µg/mL DNase I (Sigma-Aldrich), 140 mM NaCl, 5 mM KCl, 2.5 mM phosphate buffer, 10 mM HEPES, 2 mM CaCl2, and 1.3 mM MgCl2 for 30 minutes, as described by Benedicto *et al*.^18^). Cells suspensions were then passed through a 70 µm filter and endothelial cells were isolated using mouse anti-CD31-conjugated magnetic microbeads (Miltenyi Biotec, Auburn, CA, USA) with the autoMACS Separator device (Miltenyi Biotec). Cells were plated in a 6 well plate in EC culture medium (R&D Systems, Minneapolis, MN) and were expanded and passaged using TrypLE Express (Thermo Fisher). Complement experiments were performed at passage 3.

### Complement injury

In a previous report, changes in EC behavior and cytolysis were evaluated over a range of serum concentrations (5–50%)^17^. Prior to performing the drug screen, the reproducibility and extent of cell death were evaluated after 4 hours exposure to complement intact serum compared to heat inactivated serum (56 C for 1 h with agitation every 10 min) using the PrestoBlue assay (ThermoFisher) according to the manufacturer’s instructions. Serum concentrations (Sigma-Aldrich, St. Louis, MO) of 20% and 2.5% were compared, and the Z factor^19^ and relative cell death were determined. Following the establishment of the assay, the drug screen was performed using 2.5% human serum.

### High throughput drug screen

Cells were plated in 384 well plates (Greiner Bio-One, 781091) at a density of 2,500 cells/well. Cells were allowed to attach overnight, were washed in media to remove dead or unattached ECs, and the following day cells were treated with the FDA-approved small compound library comprised of 1,080 small molecules (L1300, Selleck Chemicals, Houston Texas) on a robotic platform (Microlab STAR system, Hamilton Robotics, Reno, NV). Cells were treated with 25 µL of each compound at 2x (to a final concentration of 10 µM), were incubated at 37 C for 4 hours, followed by removal of the medium and drug, and incubation with 2.5% complement-intact human serum (Sigma-Aldrich, St. Louis, MO) for another 4 hours. PrestoBlue assay solution (Life Technologies, Carlsbad, CA) was prepared and added to each well at a final concentration of 1x (from 10x stock). Plates were incubated at 37 C for 1 hr, followed by a 30 min incubation at room temperature in the dark, and cell death was quantified using an automated plate reader (EnVision, Perkin Elmer, Waltham, MA). For the initial screen, all samples were evaluated in duplicate.

### Replication studies

Two compounds found to reduce cell death in the initial screen, econozale nitrate and miconazole nitrate (Sigma-Aldrich, St. Louis, MO) were further studied in a hypothesis dependent fashion.

Protection from cell death was validated by pretreating cells in 96 well plates with econozale nitrate, miconazole nitrate, or vehicle alone (1% methanol or DMSO, respectively) followed by exposure to serum. Cell death and viability were quantified using PrestoBlue (Thermo Fisher) and lactate dehydrogenase using the Pierce LDH Cytotoxicity kit (Thermo Scientific, Waltham, MA) kit as described previously^17^.

Primary mouse choroidal endothelial cells (1 × 10^5^) were plated onto 96 plates until they reached 95% confluency. Activation of complement was confirmed as described previously for RF/6A cells^17^. Briefly, cells were passaged onto an 8 well chamber slide and were incubated with 20% human serum or 20% heat inactivated serum for 4 hours, followed by fixation in 4% paraformaldehyde and detection of the membrane attack complex with anti-MAC monoclonal antibodies (Dako clone aE11, Agilent, Santa Clara, CA). Cells were incubated for 4 hours either without drug or with 50 µM econazole or 50 µM miconazole, prior to treatment with 5% serum for 4 hours. Controls included cells exposed to serum without drug pretreatment or cells not exposed to drug or serum (n = 3 per group). Presto blue and LDH assays were performed as described above.

### Measurement of membrane attack complex

RF/6A cells were plated on 96 well plates at a density of 6 × 10^4^ cells/well. Cells were pretreated with econazole nitrate (50 µM, n = 3 wells), myconazole nitrate (50 µM, n = 3 wells) or vehicle for 4 hours, followed by exposure to complement-intact serum for 4 hours. Wells were then fixed in 4% paraformaldehyde for 15 minutes and were then labeled with antibodies directed against the neoepitope of complement C9 within the membrane attack complex, as described previously^17^. Fluorescence was quantified using a plate reader with excitation of 488 nM and emission of 520 nM (Alexa-488 dye), and excitation of 358 nM and emission of 461 nM, DAPI.

### Exosome quantification

In order to assess the extent to which the identified drugs affect the plasma membrane, we sought to determine whether treated RF/6A cells alter their exosome release. 5 × 10^5^ cells were plated in 6 well plates. When cells reached 95% confluency, they were treated with econazole nitrate (50 or 100 µM) miconazole nitrate (50 or 100 µM) or vehicle alone for 4 hours. Samples were run in triplicate. Conditioned media was then collected and exosomes were quantified using the ExoQuick-TC kit (SBS Biosciences, Palo Alto, CA) according to the manufacturer’s instructions. In addition, exosomes were quantified in drug- or vehicle-treated cells that were subsequently exposed to 5% complement-intact serum for 4 hours.

### Endosome characterization

RF/6A cells were cultured in 8-well chamber slides at a concentration of 40,000 cells per well and were cultured for 48 hours. Cells were divided into the following treatment groups: vehicle alone, 10 µM econazole, 50 µM econazole, 10 µM miconazole, and 50 µM miconazole, with 3 replicates for each treatment. The cells were treated with econazole, miconazole, or their respective vehicles for 4 hours, followed by removal of the medium and replacement with 200 µL of serum free DMEM. 12 µL/well of the baculovirus Rab5a-GFP early endosome labeling reagent was added to each well(CellLight® Early Endosomes-GFP, BacMam 2.0, Life Technologies, Eugene, Oregon)^20^. Cells were incubated overnight, fixed with 4% paraformaldehyde, permeabilized with 0.1% Trition X-100 and labeled with diamidino-2-phenylindole. Random fields were photographed using epifluorescence masked to treatment condition. All photomicrography was performed under identical exposure and gain settings. In additional experiments, fluorescence of Rab5a-GFP was quantified on a plate reader.

### Evaluation of cell surface characteristics following complement injury

Cells were plated on 6 well, TC-plates (Sarstedt, Numbrecht, Germany) at a confluency of approximately 15% and were incubated with 5% serum, either after incubation with vehicle alone (1% methanol) or after incubation with 50 µM econazole in vehicle for 4 hours. After adding serum, cells were recorded using a Holomonitor M4 laser microscope (Phase Holographic Imaging, Lund, Sweden.) Images were recorded every 10 minutes for 24 hours. The average cellular roughness (an estimate of the difference in cell surface topography between the theoretical rounded cell and the actual pixel values) was quantified in all treatments using Hstudio M4 software (version 2.7.1).

### Statistical analyses

For comparisons of cell phenotypes in drug vs. control treatment groups, all experiments used at least 3 replicates. Figures show average values and error bars show the standard deviation. A two-tailed Student’s t-test was used to determine significance, with p < 0.05 considered significant.

## Results

### Econazole and miconazole protect EC from cell death

Of 1,080 screened compounds from a panel of FDA approved drugs (L1300, Selleck Chemicals, Houston Texas), a total of 149 were associated with cell rescue with 50% or higher cell survival compared to the average of all compounds. Of these 149, 3 met our criteria and replicated across duplicate plates. Two of these, econazole nitrate (Ecz) and miconazole nitrate (Mcz), belong to the same family of compounds (imidazoles) with very similar structure, differing by the substitution of one chlorine atom (Fig. 1), and these were selected for further study.

**Figure 2:**
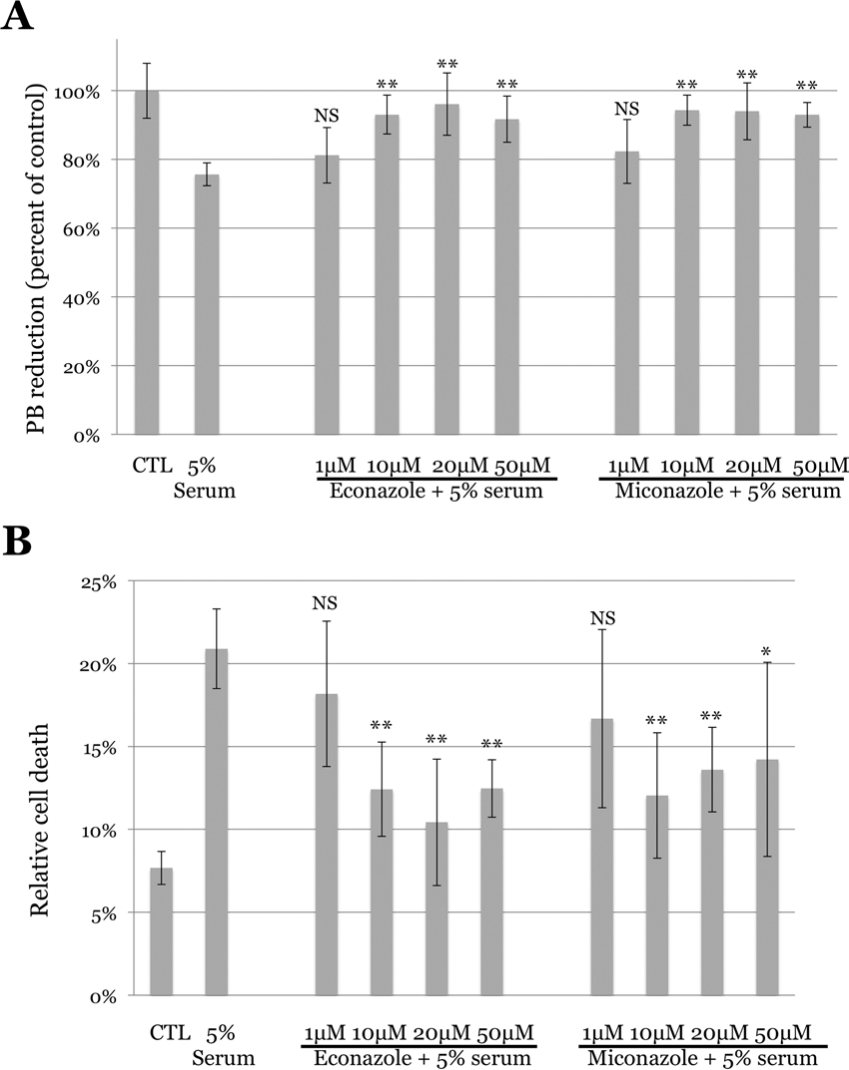
Replication of econazole nitrate and miconazole nitrate as protective compounds from complement injury. Endothelial cells were exposed to 1 to 50 µM econazole nitrate (Ecz) or miconazole nitrate (Mcz) for 4 hours, followed by challenge with 5% complement intact human serum for 4 hours. Both Ecz and Mcz showed increased cell viability according to the PrestoBlue (PB) reduction assay (**A**) and that both compounds decreased cell death according to the lactate dehydrogenase (LDH) assay (**B**) at 10, 20 and 50 µM concentrations. NS, not significant compared to 5% serum alone; *p < 0.05; **p < 0.01. Error bars indicate the standard deviation.

In validation experiments, pretreatment with either Ecz or Mcz showed a significant decrease in complement-mediated cell lysis. Using the PrestoBlue reduction assay, 5% complement intact serum caused significant suppression of reduction. Pretreatment of cells showed protection with 10, 20 and 50 µM Ecz and Mcz. At 1 µM, Ecz and Mcz showed a trend toward restored reduction but did not reach statistical significance (Fig. 2A). Similarly, when cell death/permeability (relative to 100% lysis with Triton X-100) was quantified, 5% complement intact serum showed increased cell death that was prevented by both drugs at 10, 20 and 50 µM concentrations, but not at the 1 µM concentration (Fig. 2B).

### Drug pretreatment does not significantly reduce MAC activation

Evaluation of anti-C9 immunoreactivity was performed on a fluorescent plate reader using a range of vehicle or Ecz or Mcz doses (0.5 to 150 µM) and serum concentrations (5–20%). Drug pretreatment did not consistently result in decreased immunofluorescence (data not shown).

### Miconazole reduces exosome release from RF/6A cells in a complement-independent fashion

**Figure 3:**
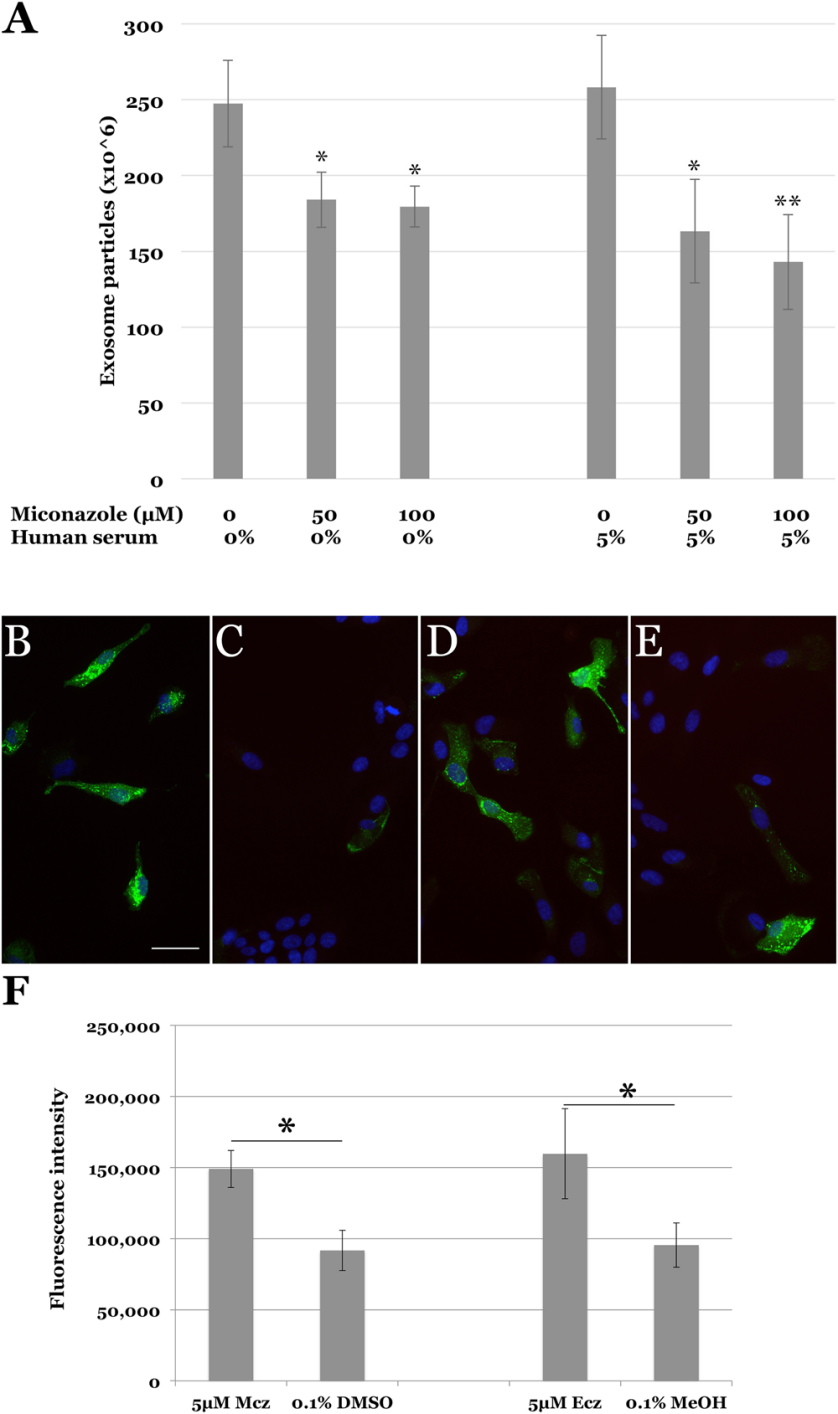
Effects of imidazole drugs on endothelial exosomes and endosomes. (**A**) Exosome release is decreased by miconazole nitrate treatment, both independently (left) and after treatment with 5% complement intact serum (right). Increased abundance of Rab5a-positive endosomes following 4 hours treatment with 50 µM miconazole (**B**) miconazole vehicle (**C**), 50 µM econazole (**D**), or econazole vehicle (**E**). This increase was also observed in both miconazole and econzaole treatment when quantified by fluorescence (**F**) *p < 0.05; **p < 0.01; scalebar in B = 50 µm.

Exosome release was quantified in conditioned media using the ExoQuick-TC kit. The curve from quantification of provided standards showed a good fit (r^2^ = 0.99). Compared to treatment with vehicle alone, treatment of cells with 50 µM and 100 µM Mcz reduced exosome release by 25% and 27%, respectively (p < 0.05, (Fig. 3A). Pretreatment with Mcz followed by 5% serum showed reduction of detected exosomes by 37% and 45% for 50 µM and 100 µM miconazole, respectively (Fig. 3A). Econazole was assessed under the same conditions, and showed a more modest impact on exosome release that was statistically significant only in the 5% serum-treated group (data not shown).

### Econazole and Miconazole increase endosome generation

Incubation of cells with the baculoviral transfection endosomal labeling kit, which transduces mammalian cells with a transgene encoding a Rab5a-GFP fusion protein, shows the presence and abundance of endosomes. Cells treated with either miconazole or econazole at 5 µM or 50 µM concentrations showed a dramatic increase in endosome abundance compared to untreated or vehicle only controls (Fig. 3B–E), both in the number of positive cells and the fluorescent intensity of Rab5a-containing endosomes. Quantification using a fluorescent plate reader showed significantly increased Rab5a-GFP fluorescence compared to vehicle treated wells (Fig. 3F).

### Drug pretreatment with econazole alters complement induced cell surface roughness

**Figure 4:**
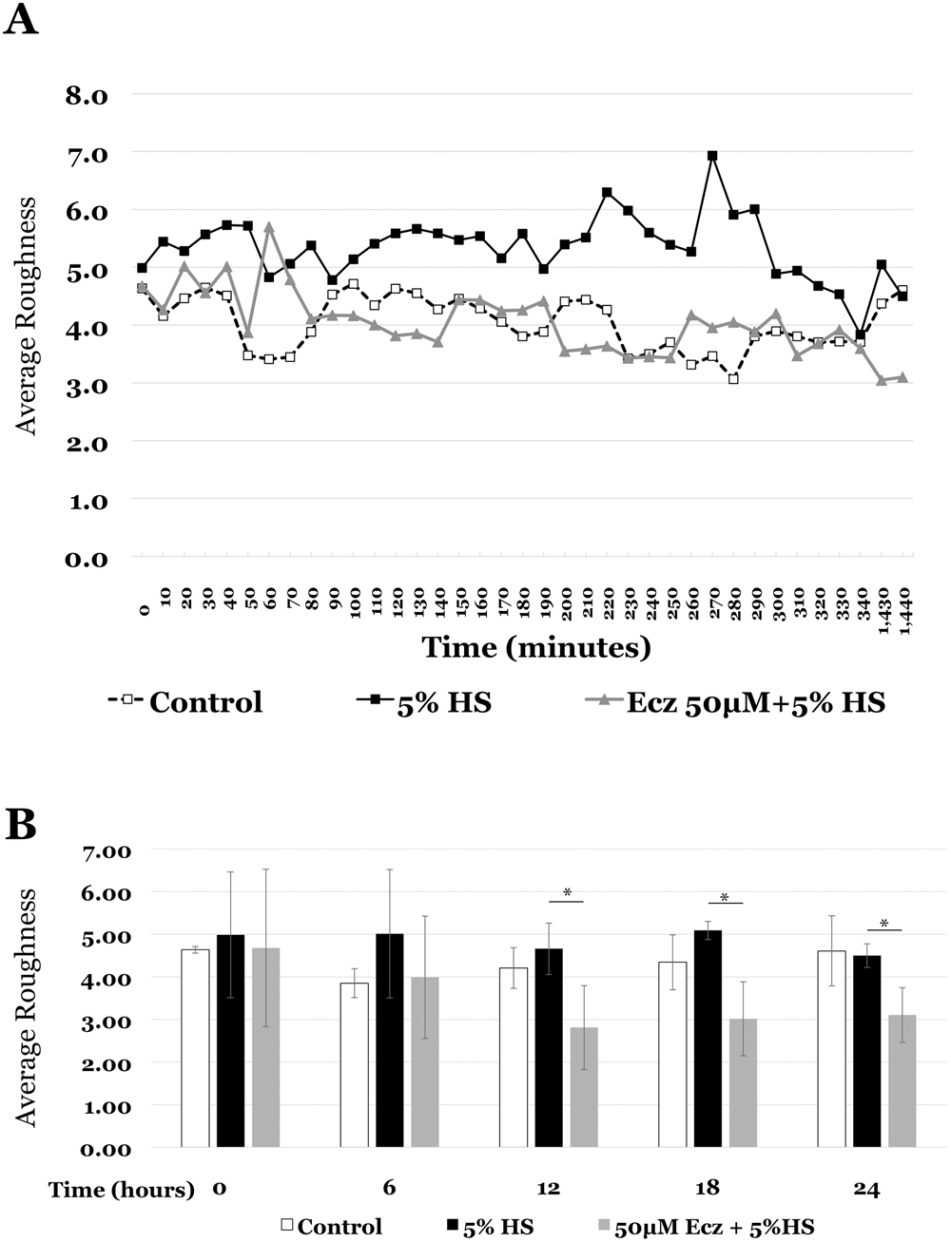
EC show increased cell surface roughness with MAC treatment that is decreased by econazole (Ecz) pre-treatment in a time dependent fashion. (**A**) Roughness measurements over 24 hours, with data sampling every 10 minutes. Exposure to 5%, complement intact human serum (5%HS, black squares) resulted in increased cell surface roughness that increased over the course of the experiment, compared to control cells (buffer only, open squares). Econazole (Ecz) pretreatment (gray triangles) reduced the 5%HS-induced increased cell roughness. (**B**) Average cell roughness at 6 hour intervals was compared between the Ecz-pretreated and 5%HS cells. Notably, the roughness at 12, 18 and 24 hrs was significantly lower in Ecz pretreatment (*p < 0.05).

Cell surface features were quantified using a holomonitor M4 laser microscope system. Cellular “roughness” is a measurement of cell surface topography and is estimated as the difference between the mathematically rounded cell surface and the actual recorded position of each pixel (http://www.phiab.se/reports/manuals/M4-SetupAndOperationManual.pdf). Cells treated with complement containing serum showed increased roughness compared to untreated controls, whereas cells exposed to econazole for 4 hr prior to serum challenge showed significantly decreased roughness compared to serum treatment alone over 24 hours of observation, particularly after the first 12 hours (Fig. 4A,B).

### Econazole and Miconazole protect primary choroidal EC from complement mediated cell death

**Figure 5:**
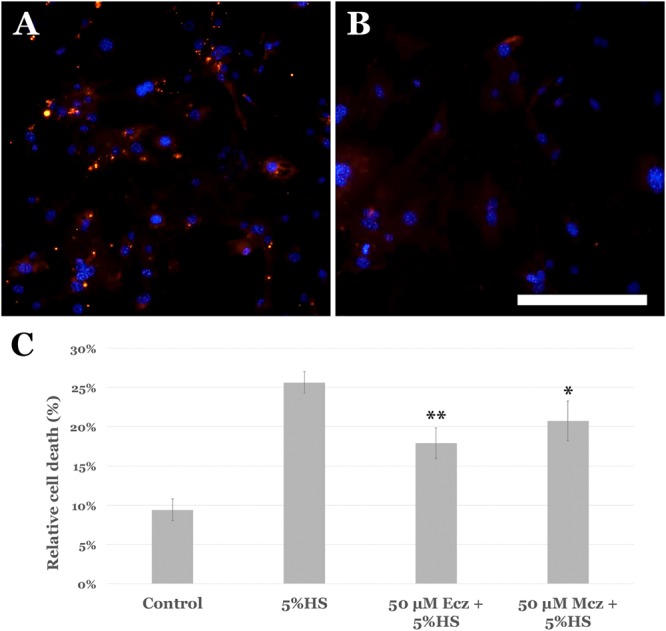
Protective effects of miconazole and econazole on primary cultures of choroidal endothelial cells. (**A**) Murine choroidal endothelial cells show activation of the membrane attack complex when exposed to serum (**A**) but not control medium (**B**), see also^17^. Pretreatment of cells with econazole (Ecz) and miconazole (Mcz) shows protection from complement induced cell death on lactate dehydrogenase assay (*p < 0.05; **p < 0.01).

In addition to an immortalized endothelial cell line, we sought to determine whether primary cultures of choroidal endothelial cells were protected from MAC injury following Ecz/Mcz pretreatment. In primary cultures of murine choroidal endothelial cells, incubation with complement-intact human serum led to deposition of immunocytochemically detectable MAC on the EC surface (Fig. 5A) that was absent from control cells (Fig. 5B). Pretreatment of mouse choroidal ECs for 4 hours with econazole nitrate or miconazole nitrate, each at 50 µM, resulted in significantly increased survival based on the LDH assay (Fig. 5C).

## Discussion

Human eyes with AMD show significantly elevated levels of the membrane attack complex surrounding choriocapillaris vessels and also show loss of choroidal endothelial cells with advancing disease. Since choroidal endothelial cells are susceptible to complement mediated lysis *in vitro*, we postulate that the membrane attack complex is involved in the degeneration of choriocapillaris EC observed in AMD patients. We therefore sought to identify compounds that protect choroidal EC from complement induced injury. The large battery of drugs screened revealed a protective effect of two very closely related compounds that reproducibly showed rescue from cell death.

Econazole and miconazole are imidazole compounds with potent antifungal properties, and that differ from each other by one chlorine atom^21^. Both drugs are commonly employed antifungals that are delivered topically for dermal or vaginal fungal infections as an ointment or cream, but both can also be applied orally, and econazole is well tolerated in an eyedrop formulation with cyclodextrin^22^. Moreover, intravitreal injection of other miconazole and ketoconazole at substantially higher doses do not result in retinal toxicity^23,24^.

Pretreatment of EC with econazole and miconazole, while resulting in decreased MAC-mediated lysis, did not appear to significantly reduce the formation of the MAC as quantified on the EC surface. We therefore suspect that these drugs act by altering the cell surface rather than inhibiting MAC formation. Imidazole compounds have a range of biological effects, that include impaired calcium entry^25,26^, inhibition of cytochrome P450 mediated prostaglandin synthesis^27^, and, in protists and fungi, interference with ergosterol synthesis^28^.

In light of the effects of these drugs on the plasma membranes of pathogens, it is possible that imidazole compounds affect membrane dynamics. In addition to the numerous molecular steps involved in interference with MAC formation, one can readily envision at least two mechanisms whereby the critical barrier function of the endothelial cell membrane could be restored after compromise by the MAC: regions of MAC-ridden membrane could be either jettisoned in an exocytic process or, alternatively, could be recovered by endocytosis. Both of these processes arrest fluid and calcium exchange across a damaged plasma membranes and both have been observed, with the degree of exocytic and endocytic activation varying depending on the cell type^29,30^. We hypothesized that exposure to miconazole and econazole may exert protective effects on endothelial cells by elevating release of MAC laden exosomes into the medium. Instead, drug treatment led to decreased exosome release, more consistent with an endocytic mechanism. Indeed, endosome trafficking was increased in the presence of miconazole and econazole (Fig. 3B).

The possibility that altered membrane dynamics, shown both by decreased surface roughness and increased endosome trafficking in this study, results in differential sensitivity to MAC injury by ECs is consistent with a recent report that membrane stiffness (associated with cellular senescence) contributes to degree of MAC injury^31^. Further studies will be required to determine specific molecular steps involved in this process and how those steps are impacted by imidazole drugs.

Inhibition of the complement system in the aging macula, either at initiation, amplification, or terminal phases, is a major area of focus for slowing or arresting the progression of this disease (e.g.^32–37^). The results in the current study provide further proof of principle that small molecules can protect choroidal cells from MAC-induced cell death and suggest that FDA approved compounds may be beneficial in reducing vascular loss and progression of AMD.
